# Asian Carp, an Alternative Material for Surimi Production: Progress and Future

**DOI:** 10.3390/foods11091318

**Published:** 2022-04-30

**Authors:** Manatsada Yingchutrakul, Naphat Wasinnitiwong, Soottawat Benjakul, Avtar Singh, Yanyan Zheng, Elliot Mubango, Yongkang Luo, Yuqing Tan, Hui Hong

**Affiliations:** 1 Beijing Laboratory for Food Quality and Safety, College of Food Science and Nutritional Engineering, China Agricultural University, Beijing 100083, China; y.manatsada@gmail.com (M.Y.); naphatnern@gmail.com (N.W.); mubangoe@gmail.com (E.M.); luoyongkang@cau.edu.cn (Y.L.); yuqingtan@cau.edu.cn (Y.T.); 2 International Center of Excellence in Seafood Science and Innovation, Faculty of Agro-Industry, Prince of Songkla University, Songkhla 90112, Thailand; soottawat.b@psu.ac.th (S.B.); avi2620@ymail.com (A.S.); 3 Institute of Agri-Food Processing and Nutrition, Beijing Academy of Agriculture and Forestry Sciences, Beijing 100097, China; zhengyanyan@iapn.org.cn; 4 Center of Food Colloids and Delivery for Functionality, College of Food Science and Nutritional Engineering, China Agricultural University, Beijing 100083, China

**Keywords:** Asian carp, surimi process, gelation, surimi additive, surimi product

## Abstract

Asian carp is a general designation for grass carp, silver carp, bighead carp, and black carp. These fish species belong to the family *Cyprinidae*. In 2018, more than 18.5 million tons of Asian carp were produced globally. Asian carp can be used for producing surimi, a stabilized myofibrillar protein concentrate that can be made into a wide variety of products such as imitation crab sticks, fish balls, fish cakes, fish tofu, and fish sausage. Surimi is usually made from marine fish, but Asian carp have been widely used for surimi production in China. The quality of surimi is affected by various factors, including the processing methods and food additives, such as polysaccharides, protein, salt, and cryoprotectant. With an impending shortage of marine fish due to overfishing and depletion of fish stocks, Asian carp have a potential to serve as an alternative raw material for surimi products thanks to their high abundancy, less emissions of greenhouse gases from farming, desirable flesh color, and sufficient gel forming ability. The utilization of Asian carp in surimi production could also contribute to relieving the overflow of Asian carp in the United States.

## 1. Introduction

Surimi is a food product widely found in East Asian cuisines, which comes in many shapes and sizes, from fish balls to various kinds of seafood imitation, such as crab sticks. Surimi is made from deboned, minced, and washed fish meat. To be able to imitate the texture of other seafood products, a good gelling property is among the most important characteristics of high-quality surimi. In surimi gelation, myofibrillar proteins, which consist of myosin and actin, play an important role. Although myosin alone can form the gel, actin also cooperates in gelation, which is influenced by the actomyosin ratio. In gelation, heating induces the denaturation of myofibrillar proteins followed by an irreversible aggregation and is cross-linked to form a three-dimensional network [1,2]. 

Nowadays, there is a wide variety of fish that is currently used as a raw material in surimi production and the majority of them are marine fish. Among those fish species, Alaska pollock is a typical commercial fish used for surimi production as a raw material. It is a cold-water white fish that is available in the North Pacific [3]. The superior gel properties, white flesh, and its availability make it suitable for surimi making. Apart from Alaska pollock, there are several cold-water white fish that are also used in surimi manufacturing, which includes the Arrowtooth flounder, Pacific whiting, and blue whiting [4,5,6,7]. In tropical countries, such as countries in Southeast Asia, tropical fish including threadfin bream, bigeye snapper, and lizardfish are some examples used to produce surimi and surimi products [4]; however, overexploitation of these lean fish species diverts the interest of surimi processing industries towards dark fleshed fish called pelagic fish including sardine, mackerel, etc., but these fish possess weak gelling properties, which are associated with the high lipid, water-soluble protein contents and endogenous proteases firmly attached to the fish muscles [8,9]. Therefore, to overcome the aforementioned problems, freshwater fish could be used as a raw material for surimi production. These freshwater fish have already been used in traditional surimi products, such as fish balls and fish cakes, in China [10]. 

In general, freshwater fish can be cultured at a low cost and they can attain optimum size in a short time. Therefore, they are gaining more attention to be considered as a potential candidate for surimi manufacturing. Asian carps, namely, bighead carp, grass carp, silver carp, and black carp, are freshwater fish, which can be commonly found in Chinese cuisine. Various recent researches have been performed to improve the gel properties of surimi made from Asian carp [11,12,13,14], and although various collective reports have been available on lean and dark flesh fish surimi gel, no review has been available on the usage of Asian carps in surimi gel preparation and factors affecting gelation. Therefore, this review aims to promote a comprehensive understanding of the Asian carps’ characteristics and their suitability for surimi production. Moreover, factors affecting and solutions to improve the qualities of surimi from Asian carp have also been revisited.

## 2. Surimi Material Resources

Surimi processing technology was established in the 1960s. At that time, cold-water white fish was used as the main material for surimi production, especially the Alaska pollock; however, the market for surimi and surimi products has continually developed and grown in subsequent years, and various marine fish species have been utilized as a material in the surimi industry such as Arrowtooth flounder, Pacific whiting, threadfin bream, and bigeye snapper [4]. The fish materials for surimi production differ from area to area depending on several factors including ecosystem productivity, fish intensity, management, price, and the quality properties of products.

Nowadays, overfishing and depletion of marine fish stocks are becoming a major concern for the fisheries industry as well as the surimi manufacturing industry. With a decreasing supply of marine fish, Asian carp as an alternative ingredient could be exploited. Asian carp can be produced in a large quantity in a shorter time and at a lower cost as compared to marine fish. The amount of grass carp, silver carp, bighead carp, and black carp produced worldwide in the year 2019 were 5.7, 4.7, 3.1, and 0.7 million tons, respectively. On the other hand, 3.5 million tons of Alaska pollock, 0.4 million tons of pacific whiting, and 0.03 million tons of Arrowtooth flounder were produced in the same year [3]. Moreover, almost all Asian carp are currently cultured in China. Asian carp production has the potential to reach an even higher number if other countries start raising Asian carp for consumption as well. In addition to this, Asian carp also have another advantage. According to a study by Gephart et al. [15], silver carp and bighead carp farming produces the least greenhouse gas, nitrogen, and phosphorus among farmed finfish and crustaceans. This makes silver carp and bighead carp more environment-friendly choices for an alternative raw material for surimi than other farmed fish. Regarding the suitability of Asian carp for surimi production, Asian carp are a white flesh fish.

## 3. Biometric Character of Asian Carp Species

**Figure 1:**
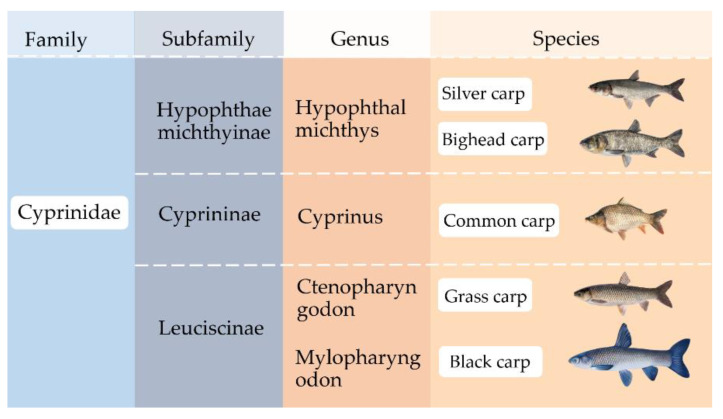
Genealogy of Asian carp.

Silver carp, bighead carp, grass carp, and black carp, have been widely consumed in Asian countries such as China and Japan for millennia. These fishes and the common carp are members of the family *Cyprinidae*, a family of freshwater fish commonly known as the carp family (Figure 1). There are about 3000 species of fish that belong to the family *Cyprinidae*, making it the largest and the most diverse fish family. It is also the largest vertebrate animal family. Asian carp species can reproduce and grow rapidly, but while this characteristic is beneficial for carp aquaculture, it can be considered a problem, and is threatening to native species and the aquatic environment in places where they are not normally consumed by the locals, such as the United States [10]. In the United States, silver carp, bighead carp, grass carp, and black carp are known as Asian carp.

Grass carp (*Ctenopharyngodon idella*), the sole member of the genus *Ctenopharyngodon*, is a member of the family *Cyprinidae* commonly found in East Asia. Grass carp can grow to a maximum size of around 1.5 m [16]. The main diets of grass carp are aquatic plants. Grass carp is the most produced Asian carp, as well as the most produced fish in aquaculture worldwide and there was 5.7 million tons of grass carp produced in 2018 [3]. Grass carp has white flesh which is composed of 16.70% protein and 0.12% fat [10].

Silver carp (*Hypophthalmichthys molitrix*) belongs to the genus *Hypophthalmichthys* which can grow to 1 m in size [16]. The silver carp is an omnivore but feeds mainly on phytoplankton. Silver carp is produced more than other fish species worldwide in aquaculture, second only to the grass carp. According to the Food and Agriculture Organization [3], more than 4.7 million tons of silver carp were produced in 2018, compared to 5.7 million tons of grass carp produced in the same year. Silver carp also has white flesh and is high in protein (15.65%) and low in fat (1.89%) [10].

Common carp (*Cyprinus carpio*), also known as European carp, is a carp species native to Asia and Europe. Common carp have an average size of 40–70 cm, but can grow up to 120 cm [17]. Similar to other carp species, common carp are omnivorous but prefer insects and crustaceans. In the year 2018, 4.3 million tons of common carp were produced globally [3]. Common carp flesh is composed of 15.4% protein and 1% fat [10].

Bighead carp (*Hypophthalmichthys nobilis*) belongs to the genus *Hypophthalmichthys*, the same genus as silver carp. The bighead carp’s maximum size is around 1.5 m [16]. The bighead carp is an omnivorous filter feeder that prefers zooplankton as its main diet. Bighead carp is less produced compared to silver carp and around 3.1 million tons of bighead carp were produced worldwide in 2018 [3]. The bighead carp’s flesh is white and firm, and it contains 17.90% protein and 0.96% fat [10].

Black carp (*Mylopharyngodon piceus*) is the only member of the genus *Mylopharyngodon*. The average size of a black carp is not so different from that of the other Asian carp species. Its maximum size is 1.2 m [16]. The black carp’s main diet is mollusks, such as clams and snails. Only 0.7 million tons were produced in 2018, which is the lowest among the other Asian carps [3]. The black carp’s flesh consists of 17.06% protein and 8.58% fat [10], which is the highest fat content compared to other Asian carp.

According to the information mentioned above. Asian carp species, namely, silver carp, bighead carp, common carp, grass carp, and black carp, are being produced in great quantities and have a high availability. Their flesh also contains a high protein content and low fat content. Moreover, the size of Asian carp is more economical to prepare fillets for surimi production compared with the average size of threadfin bream (10–15 cm) [4]. Therefore, Asian carp has the potential to become an alternative raw material for surimi production.

## 4. Asian Carp Surimi and Surimi Products

### 4.1. Asian Carp Surimi 

**Figure 2:**
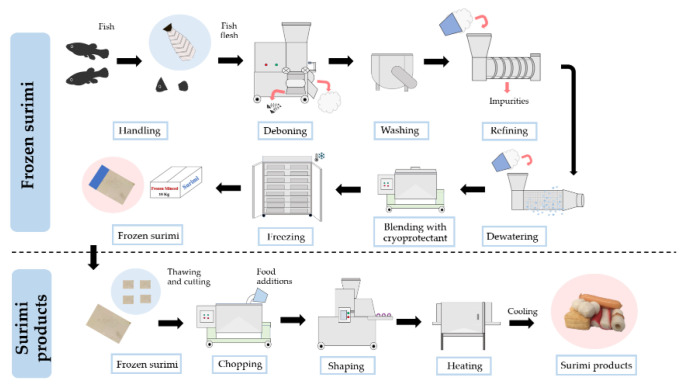
The basic processing of frozen surimi and surimi products.

Surimi is a stabilized concentrated myofibrillar protein paste that is mainly divided into two processes including frozen surimi and surimi products (Figure 2). Generally, surimi is mainly produced from lean fish, such as bigeye snapper, threadfin bream, Pacific whiting fish, etc. The high content of light muscle, and low amount of lipids in lean fish provide a high-quality gel of high whiteness [18,19]; however, overexploitation of lean fish and the lower gel-forming ability of tropical fish (Indian mackerel, sardine, etc.), means freshwater fish have been recently highlighted as an alternative material for surimi production [20]. The processing steps for surimi and surimi products from freshwater fish species are the same as the production process from marine fish species [4]. Several studies were conducted to produce surimi from Asian carp. For example, Endoo and Yongsawatdigul [21] investigated the gel-forming ability from freshwater fish including silver carp, rohu, and tilapia in comparison to marine fish (threadfin bream) surimi during frozen storage for nine months. Among the freshwater carps, the silver carp surimi presented the highest gel-forming ability; however, freshwater fish surimi showed a higher denaturation of myofibrillar proteins than those from marine fish surimi during the frozen storage. Although the gel-forming ability of Asian carp is lower than marine fish species, due to their abundance Asian carp can be used as the raw materials in the surimi industry. Consequently, various treatments including the use of food additives, washing, cooking methods, etc., have been employed to develop high-quality surimi and surimi products from Asian carp.

### 4.2. Surimi Gelation Mechanism 

A soft, firm, and elastic texture of surimi gel is produced by the gelation of myofibrillar proteins in fish muscle tissue. Myofibrillar proteins, including myosin, actin, and tropomyosin are responsible for the gelation, out of which myosin, especially myosin heavy chain (MHC), is the main component that forms an ordered three-dimensional gel network providing the gel strength and water-holding ability [22]. In general, the gelation of surimi involves the solubilization, denaturation, and aggregation of myofibrillar proteins.

Gelation of surimi occurs during the setting step. It is a step during the surimi processing where washed fish mince/surimi paste is mixed with salt and other additives to form surimi sol, which is then set at low (2–4 °C), medium (25 °C), and high (40 °C) temperatures for various setting times followed by cooking at 90 °C [23]. The optimal setting temperature and time varies with the fish species. For example, Benjakul et al. [24] reported that surimi made from threadfin bream (*Nemipterus bleekeri*), bigeye snapper (*Priacanthus tayenus),* barracuda (*Sphyraena jello*) and bigeye croaker (*Pennahai macrophthalmus*) have an improved gel quality when set at 25 °C. On the other hand, lesser sardine (*Sardinella fimbriata*) surimi can achieve a high gel strength when set at 35 °C [25]. 

When exposed to salt and heat, myofibrillar proteins will be solubilized and denatured, which in turn expose their highly reactive surfaces. Intermolecular bonds between neighboring protein molecules are formed at these sites. A 3-dimentional protein network will be formed when enough intermolecular bonds have occurred. The four major bonds that are crucial in the formation of a protein network are hydrogen bonds, ionic linkages, covalent bonds, and hydrophobic interactions.

In surimi products, the hydrogen bonds help stabilize the water in surimi gel and contribute to an increasing gel strength of surimi products. Hydrogen bonds are weak individually but occur in great number and when heated, hydrogen bonds between the carbonyl and amide groups in the peptide backbone that hold the protein folded are broken. The exposed peptide backbone then becomes highly hydrated by interacting with water in the surimi gel and, in turn, contribute to the water holding-capacity of the surimi gel [26]. Moreover, hydrogen bonds also contribute to surimi gel strength as the gel becomes cooler since more hydrogen bonds between proteins will form as the temperature drops. 

Normally, a myosin thick filament is held together by an ionic linkage. This linkage, called a salt bridge, is the most important force that keeps the myosin thick filaments together [27], which results in its water insolubility. During surimi manufacturing, salt is usually added to break the salt bridge, making the myofibrillar proteins more soluble. Moreover, salt ions also bind to the proteins’ surface and further solubilize them by increasing their affinity for water [28].

The intermolecular bonds formed via a ε-(γ-glutamyl) lysine linkage by endogenous transglutaminase (TGase) are responsible for the higher gel strength of the surimi gel [29]. This non-disulfide (a dipeptide bond) bond is formed between glutamine and lysine amino acids. A calcium ion is usually added in the form of calcium chloride in order to activate the TGase and promote the forming of ε-(γ-glutamyl) lysine bonds [30].

In addition, when the proteins are unfolded, their inner part consisting of hydrophobic amino acids is exposed to water molecules and these hydrophobic parts of the proteins tend to clump together similarly to oil or fat droplets in water to minimize their exposure to water. This attraction between the hydrophobic parts of proteins gives a similar result to the binding of proteins. Thus, it can lead to protein aggregation and the formation of a gel network.

### 4.3. Traditional Asian Carp Surimi Products

Surimi products have been developed in several styles by a combination of various shaping and cooking methods. Asian carp surimi has been used to produce various kinds of traditional surimi products, such as fish cake, fish balls, fish tofu, and fish sausage. These products are ready-to-eat foods, rich in protein, low in fat and have an improved texture and taste.

Fish cake, a traditional Chinese cuisine, is produced by various heating methods including steaming, boiling, and frying. The texture and shape of the cake are different, depending on the geographical region [31]. Chinese fish cake is mainly prepared with a surimi paste mixed with starch, egg white, soy protein, or other ingredients, shaped into oval or ball shapes, followed by frying. The final products of fish cake are characterized by a crispy outside and tender or soft inside. Lu et al. [32] optimized the formulation of fish cake from silver carp, which consist of 54% surimi paste, 10% pig fat, 12% cornstarch, 12% moisture, 6% albumin, 2% salt, 2% sugar, 1% cooking wine, 0.5% ginger, and 0.5% monosodium glutamate. The mixture was then subjected to steaming for 20 min.

In addition to the fish cake, fish balls are one of the most famous traditional foods in Southeast Asia, especially in China. According to the literature, fish balls occurred during 530 the Qin Dynasty (BC 221-207) [10]. Various Asian carp species such as the silver carp, common carp, and grass carp, have been utilized for fish ball production [33,34,35]. Fish balls are mostly served with soup-based dishes, noodle-based dishes or fried. The basic process includes the thawing of frozen surimi, chopping, mixing, setting gel, cooking, cooling, freezing, frying, cooling after frying, packaging, and storage. The main ingredients of fish balls include sugar (3%), salt (3–5%), starch (3%), monosodium glutamate (1%), and water (40%) [36]. To determine the quality of fish balls, the flotation method has been used. In general, the ability of surimi to sink or float in water determines its quality, in which the sinking of balls is considered a deformation. To prevent the deformation of fish balls, salt is added at the last addition for keeping the extruded fish balls floating in the middle of the water [37]. In addition, unwashed fish balls have been considered to contain more nutritional value. Moreover, the production has a reduced water consumption and wastewater disposal in the washing process [38]. 

Fish tofu is widely consumed as a convenient snack food or served with a hotpot. Fish tofu is a fish-based emulsion formulation that is made from fish mince or surimi as the main ingredient, chopped and then mixed with food additions such as soybean oil (4.50%), soybean flour (8.50%), tapioca flour (4.50%), egg white (12.50%), ice (12.70%), sugar (2.4%), and salt (1.20%). For the cooking step, commercial fish tofu generally uses the frying method before packing and storage [39]. The main method of cooking tofu is deep-fat frying at high temperatures. The fish tofu has a golden brown surface, and tender mouthfeel, with a slightly fishy smell and unique frying flavor; however, the frying process could increase the oil content of the products. Nowadays, the trend of healthy food has influenced every part of the food industry, therefore, the demand for low-fat foods is increasing day by day. Previous studies have established that the vacuum frying process (120 °C at 21 kPa) is an alternative frying method for reducing the final content of oil in fish tofu [40,41]. 

Sausage is a traditional product that can be prepared from various meats such as pork, chicken, beef, and fish. The sausage manufactured from fish is an alternative source of high protein and low-fat content [42]. Various Asian carp species such as silver carp and common carp, have also been interested in sausage production [43,44]. Moreover, fish sausage has been popularly consumed as a convenient snack food. Fish sausage could be produce by a basic step process that includes the thawing of frozen surimi, cutting, mixing with food additives, stuffing, lipping casing, inspecting pinholes, and sterilization [45]. In addition, fish sausage also has been applied to a variety of snacks by adding cheese or various seasonings such as a spicy flavor or barbecue flavor [10]. 

In addition, fish noodles are also a traditional product with a long history. Previous studies have utilized Asian carp species for noodles production such as grass carp, silver carp, and bighead carp [46,47,48] and these were prepared from surimi paste. Fish noodles are commonly served with soup-based dishes, in which a variety of ingredients such as fish balls, vegetables, and fish flesh have been added.

## 5. The Quality Improvement of Asian Carp Surimi and Surimi Products

Quality characteristics of surimi are the main concerns for the determination of surimi quality. Several factors have been studied and applied to improve the quality of surimi, such as in the raw materials (e.g., species, harvesting seasons, rigor mortis, freshness and off-odor), processing methods (e.g., washing treatments, cooking methods), and functional ingredients (e.g., polysaccharides, protein, microbial TGase, salts, cryoprotectants, polyphenols, and oils).

### 5.1. Raw Materials

Fish species, harvesting season or conditions and the freshness of the fish mince affect the quality and characteristics of surimi as well as surimi products. Yuan et al. [49] reported that the thermostability of fish myofibrillar proteins is also varied among fish species as well as in their cultured conditions. They reported a different thermal stability (as determined by the inactivation rates of myofibril ATPase) of myofibrillar proteins extracted from fish raised under different seasons (summer and winter). For example, surimi from silver carp raised in the hot season (autumn and summer) required a higher setting temperature (40 °C) than those cultured in the winter season (setting temperature 30 °C), which was most likely due to the higher thermal stability of myofibrillar proteins. He et al. [50] also reported that silver carp surimi prepared in the summer season was thermally more stable than surimi made in the winter season. Moreover, the setting temperature was affected by the different seasons. Generally, myofibrillar proteins are prone to a temperature fluctuation during post-harvesting handling and storage conditions as well as time. Therefore, freshness and rigor mortis also determine the gelling ability of surimi [51]. Proper handling and storage after harvesting could lower the biochemical and microbiological changes in the raw material [52]. Protein or ATP degradation, a pH drop, protein/lipid oxidation, and production of undesirable compounds by microbes are the major changes that occur during storage, which directly affect the quality and shelf-life of fish. These changes negatively affect the texture, water-holding capacity, and color of surimi protein [53]. The mechanism of deterioration can follow four steps: rigor mortis, resolution of rigor, autolysis (loss of freshness), and bacterial spoilage [9]. The process of autolysis is mainly caused by the deterioration of fish muscle by endogenous proteinases, which could be from the digestive organs including the liver, gut, and kidney [51,53]. Gelman et al. [54] reported that the freshness of common carp after harvesting had been changed by 3–4 days of chilling storage. Lu et al. [55] reported a degradation of the myofibrillar and sarcoplasmic protein of bighead carp caused by cathepsin B and cathepsin L during the rigor mortis period. A study by Hu et al. [56] shows that adding purified cathepsin L from the dorsal muscle of carp (*Cyprinus carpio*) can drastically hydrolyze the MHC in carp surimi gel and reduce the gel’s breaking force by 24.33%. Overall, the raw material plays an important role in the production of surimi and surimi products, which is affected by the freshness, source, season, feeding habits, post-harvest-handling, etc., of the carp.

### 5.2. Washing Treatments

Washing is an important process to separate fat and undesirable materials such as pigments, blood, and odorous compounds, which result in the production of surimi with an improved gel-forming ability and whiteness. Generally, the minced fish is washed with chilled water until the removal of most of the water-soluble protein [57]. The efficiency of the washing process of surimi can be affected by various factors, such as the number of washing cycles, water volume, water/meat ratio, and washing solutions [51]. To improve the quality of Asian carp surimi, several washing solutions and washing processes, such as CaCl_2_ and pH-shifting process have been studied [58,59]. Cheng et al. [60] investigated the effects of different washing treatments (distilled water, NaCl, NaHCO_3_, CaCl_2_, and a mixture of NaCl and NaHCO_3_) on grass carp surimi. The 0.6% CaCl_2_ yielded gels had the highest whiteness and improved gel properties. Generally, Ca^2+^ ions are known to enhance the aggregation of the MHC and the formation of hydrophobic interactions during the gelation process [20]. Chang et al. [59] investigated the effects of an alkali-aided processing comparison with conventional washing of bighead carp muscle proteins. The alkali-aided processes of the surimi products had a higher muscle protein than the conventional washing process, especially in alkali-processed products. In addition, ozone was used in the surimi washing process to decrease microbial growth and muddy flavors, and to improve the whiteness of the surimi gel. Zhang et al. [61] improved the evaluated physicochemical properties as well as removed the off-flavor of bighead carp mince by ozone washing for 5–20 min. Zhang et al. [62] reported that the second cycle of ozone washing treatment (8 mg/L) resulted in a higher in whiteness of the silver carp surimi than the first cycle of the washing treatment. 

### 5.3. Cooking Methods

#### 5.3.1. Traditional Methods

A heating treatment is one of the common processes to induce surimi gel. The quality of surimi products could be dependent on the temperature, heating rate, and heating method. Temperature plays an important role in the denaturation and unfolds of the gel formation. The traditional process of surimi products has been to heat it in a two-step process by a water bath treatment; the first step heated to below 30 °C for the cross-linking of myofibrillar protein, followed by cooking at 90 °C for forming a 3-dimensional protein network and the inactivation of various indigenous enzymes [2,63]. However, water bath heating obtains a heat transfer from the outside surface to the inside of the surimi paste, and causes a slow gel-formation during the fast cooking of surimi products [64,65]. To obtain a fast cooking time with surimi products, several treatments such as microwave heating and radiofrequency have been employed [66,67]. Feng et al. [68] investigated the comparison of microwave heating (100 and 300 w) and water bath heating on the physicochemical properties of fish protein from silver carp. The results showed that the water bath treatment resulted in a surimi gel that had a higher denaturation and aggregation of actomyosin than the microwave; however, the microwave heating (300 w) showed a protective effect on the conformational change of fish protein and improved the quality of surimi. Radiofrequency results in a dielectric heating of the meat product with a low rate of electromagnetic waves. Moreover, radiofrequency has been applied in the surimi heating process to decrease the heating time and reduce nutritional losses, and to improve the quality of surimi products compared with the traditional treatment [67].

#### 5.3.2. Novel Non-Thermal Methods

In addition, non-thermal technology, an acid-induced gel, and 3D printing, have also been developed and applied to the production of Asian carp surimi products [69,70,71] (Table 1). Non-thermal technology is used as an alternative to traditional heating methods due to its minimal effect on the color, aroma, flavor, and nutrients of food products [72]. The applications of non-thermal technology in surimi products include high-pressure processing, high-intensity ultrasound, and E-beam irradiation.

High-pressure processing, also known as ultra-high-pressure processing or high hydrostatic pressure processing, is a food processing technology that utilizes a high pressure (from 100 MPa up to 900 MPa) [73]. The pressure can cause physicochemical changes and improve the functional properties of food products by enhancing the moisture–protein or protein–protein interactions [74]. The effectiveness of high-pressure processing on the quality of fish products is dependent on the amount of pressure applied and the type of product itself [75]. Liang et al. [69] compared the effectiveness of a high-pressure treatment (100, 200, 300, 400, and 500 MPa) and a two-step heating treatment (40 °C for 30 min and 90 °C for 20 min), on the gel characteristics of surimi from bighead carp. The results showed that the high-pressure treatment gave a higher gel strength and springiness compared with the traditional heating treatment, especially when 500 MPa of pressure was applied.

High-intensity ultrasound is a technique used for improving the physicochemical properties of food products, such as firmness, ripeness, acidity, and sugar content, by applying a low frequency mechanical wave (16–100 kHz) [76]. This technique can also be used for improving the gel properties of surimi products [77]. For example, Gao et al. [12] studied the effects of pre-treatment using high-intensity ultrasound on the gelation properties of surimi from silver carp. The results showed that applying a high-intensity ultrasound pre-treatment before mixing the mince with salt was the most effective pre-treatment mode for promoting the gelation of surimi. The pre-treatment increased the breaking force and prevented deformation of the surimi gel by promoting the formation of non-disulfide bonds and S–S bonds. Moreover, the efficiency of the high-intensity treatment on the gelation properties of surimi is also dependent on the salt contents [78]. 

Electron beam (E-beam) irradiation is a technique that involves utilizing high-energy electrons for a variety of applications such as pasteurization and sterilization. It is completed by shooting electrons through the product using a linear accelerator [79]. The E-beam generally uses electricity, making it safer compared to gamma ray irradiation using radioisotopes (Co^60^ or Cs^137^). The recommended dose for applying E-beam on food products is ≤10 kGy [80]. Electron beam irradiation is, however, not without flaws. Brewer [81] reported a probability that E-beam can cause off-odors and off-flavors in food products. Zhang et al. [82] reported that the application of E-beam irradiation (1–7 kGy) in combination with microwave heating (70 °C) on grass carp surimi produced more volatile compounds than an E-beam irradiation treatment alone, while the control treatment produced the least volatile compounds.

**Table 1:** The process progress to improve surimi quality.

Processing/Treatments	Name of Fish Species	Experimental Conditions	Analysis	Optimum Amount/Treatment Conditions	Reference
Acid-induced gel preparation	Silver carp	Acid-induced gel (acetic acid solution 1:4 (*w*/*v*)), and heat-induced gel (30 °C for 1 h and 90 °C for 15 min)	Moisture content, pH, TPA, expressible water content, whiteness, SDS-PAGE, SEM, and protein solubility	Acid-induced gel	[70]
3D printing	Silver carp	Printing systems; nozzle diameter size (0.8, 1.5, 2.0 mm), nozzle height (5, 10, 15, 20 mm), nozzle moving speed (20, 24, 28, 32 mm/s, and extrusion rate (0.002, 0.003, 0.004, 0.005 cm^3^/s)	Rheological characterization, gel strength, WHC, microstructure, LF-NMR, extrusion rate, and resulting diameter	Nozzle diameter 2.0 mm, nozzle height 5.0 mm, nozzle moving speed 28 mm/s, and 0.003 cm^3^/s	[71]
Non-thermal					
High pressure	Bighead carp	Heating treatment (40 °C for 30 min and 90 °C for 20 min), and pressure treatments (100, 200, 300, 400, and 500 MPa, at 25 °C for 30 min)	Gel strength, TPA, WHC, whiteness, turbidity, protein solubility, SDS-PAGE, SEM, and protein content	Pressure treatment at 500 MPa	[69]
High intensity ultrasound	Silver carp	0–5% NaCl, ultrasonic treatment at 100 kHz, 300 W for 10 min	puncture, microstructures, WHC, dynamic rheological properties, SH content, chemical interactions, solubility, TCA-soluble peptides, and SDS-PAGE	0–2% NaCl	[78]
E-beam irradiation	Grass carp	Irradiation doses; 0, 1, 3, 5, and 7 kGy in combination with microwave heating 70 °C	Volatile compounds, and fatty acid profile	5 and 7 kGy	[82]

### 5.4. Functional Ingredients

In general, to augment the gel properties, several additives such as protease inhibitors, endogenous TGase, calcium ion, phenolic compounds or cross-linking enzymes, e.g., microbial TGase (MTGase), chitooligosaccharide, hydrocolloids, etc., have been employed [83]. In this section, the functional ingredients used to improve the quality of carp surimi have been discussed (Table 2).

#### 5.4.1. Polysaccharides

Polysaccharides are known to promote the mechanical and functional properties of various gels by interacting with water and myofibrillar proteins [84]. Polysaccharides, such as starch, gum and chitosan, have been incorporated into Asian carp surimi gels.

Starch is one of the most commonly used food additives in surimi products to improve gel properties and freeze–thaw stability, as well as to replace the mince to a certain extent, which can lower the cost of the final products [85]. Generally, starch molecules are composed of amylose and amylopectin, which are connected by hydrogen bridges. The starch obtained from different sources plays different roles in the gel system [86]. For example, Liu et al. [87] reported that silver carp surimi incorporated with 8% potato starch has better textural properties than those added with 8% corn starch. This was more likely due to differences in the gelatinization conditions, granule swelling, and water-absorbing ability of both starches [88]. The gelatinization of starch has a major role in the formation of the protein–starch network structure, which is influenced by the setting conditions. In thermal processing, the starch granule could irreversibly swell, absorb water, and change the viscosity, concomitantly with the thermal gelation of myofibrillar proteins [87,89]; however, the starch granules could absorb water into a small fraction of the gel matrix [89]. Wu et al. [90] studied the addition of starches on grass carp and silver carp surimi that had a lower breaking force than non-starch surimi when the starch concentration increased. Wang et al. [91] reported the addition of surimi (10% and 30%) in combination with wheat flour dough, that required more heating time during microwave treatment to achieve the targeted temperature. In contrast, increasing starch concentrations resulted in brittle gels with poor gel properties [92]. This could be due to the competition for water between protein and starch molecules. In addition, modified starches are also used in the surimi industry because of their improved functional properties over native starch. Modified starch can improve shelf-life, the freeze–thaw stability, and storage stability of surimi products [93,94]. Cao et al. [95] reported the addition of modified starch-sucrose mixtures (1:1, 8%; *w*/*w*) to silver carp surimi that prevented the conformational changes of actomyosin during frozen storage for 90 days.

Gums are described as complex polysaccharides which have been found from various plant sources, such as plant seeds and plant exudates [96]. Gums have been used as a food additive to improve the function and texture of surimi products which could increase the yield of a product without negative effects on the color and texture [84]. Moreover, they also improve the water-holding properties of surimi due to their good water absorptivity [97]. Xiong et al. [97] reported that with an increasing konjac glucomannan addition, the breaking force, deformation, and water-holding capacity of surimi from grass carp were enhanced. Mi et al. [98] reported that a combination of 1.37% hydroxypropylated cassava starch (HCS), 0.44% curdlan (CD), and 0.22% κ-carrageenan (KC) had significant effects on the microstructure of silver carp surimi compared to those with starch or gum alone added, which was confirmed by the compact and ordered network structure of the former as compared to the latter one. Barrera et al. [99] investigated the effect of a 1% pectin gum and 0.2% CaCl_2_ combination with different degrees of methoxylation (27–35%, 27–33%, 60%, 65%, 69–72%, and 72%). The results showed that silver carp surimi that was supplemented with a low methoxyl (27–33%) of pectin gum in combination with CaCl_2_ had an improved shear stress, hardness, and water-holding capacity. Buda et al. [100] investigated the effects of a konjac glucomannan combination with apple pectin on the gel properties of silver carp surimi. The results showed that the addition of 0.025% apple pectin in combination with 2% konjac glucomannan successfully improved the gel-forming ability as indicated by a higher gel strength. 

Chitosan is a deacetylated derivative of chitin which has been found in marine invertebrates, insects, fungi, and yeasts [101]. In particular, the applications of chitosan in food products were aimed to enhance antioxidant properties, film-forming ability, and gelling properties [102]. Li and Xia [103] investigated the effects of chitosan with different molecular weights (299, 410 600, 706, and 880 kDa) and deacetylation (60.5%, 65.4%, 70.8%, 77.3%, 86.1%) on salt-soluble protein meat from silver carp. The results showed that chitosan (880 kDa) with a degree of deacetylation of 77.3% had the highest penetration force, and storage modulus in salt-soluble protein meat from silver carp. Wu and Mao [104] reported that the addition of 1% chitosan with a mixture of molecular weight (300 and 10 kDa) to a kamaboko gel prepared from grass carp surimi showed higher effects in the gel during storage for 15 days at 4 °C, which was more likely associated with the antioxidant and antibacterial activity of chitosan.

In addition, some fiber additives such as nanosized okara, seaweed, and chicory root, have also been employed to improve the gel properties of Asian carp surimi [105,106,107]. 

#### 5.4.2. Protein Additives

Proteins such as egg white, soy protein isolates, and whey protein concentrate have been widely used as additives for gel strength improvement, as a proteinase inhibitor, and for the anti-retrogradation of starch during storage. Moreover, some protein additives have been employed to enhance the color and flavor of gel products [108]. 

Egg white powder (EWP) or liquid is the most widely employed protein additive in food products due to its wide functional properties, such as emulsification, foaming, gelation, adhesion, etc. [109]. EWP acts as a protease inhibitor, which can inhibit gel weakening (associated with the endogenous muscle proteases) during cooking. Moreover, it can act as a gel filler and has the ability to improve the whiteness and textural properties of surimi gel [110]. Walayat et al. [111] indicated that a mixture of 6% EWP and β-cyclodextrin improved the structural and functional properties of myofibrillar protein from silver carp during frozen storage as indicated by a compact, dense, and network structure of the gel. 

Soy proteins are generally derived from defatted soy flakes, as soy protein concentrate and soy protein isolate. Soy proteins are one of the lowest cost food additions used as flavoring, nutritional, and functional additives in surimi products [108]. Moreover, they also contain protease inhibitors, especially serine-proteases [112]. Luo et al. [113] reported that the addition of 10% soy protein isolate in silver carp surimi inhibited the modori phenomenon, which is a major cause of the degradation of myofibrillar proteins during heating. The efficiency of soy protein isolate on the quality of surimi can be affected by various factors, such as setting conditions (time and temperature), and protein concentrations [114,115]. Luo et al. [116] investigated the effects of the different concentrations of soy protein isolate (0–40%) at different setting conditions on the textural properties and microstructures of bighead carp surimi. The results showed that the addition of 10% soy protein isolate and heating at 35 °C to 40 °C for 60 min could effectively enhance the gel strength of bighead carp surimi. 

Whey protein concentrate (WPC) is a by-product of dairy manufacturing. WPC molecules are the components between two main proteins, β-lactoglobulin and α-lactoalbumin. It has been used for a variety of applications such as for protein supplements, foaming stabilizers, water binders, thickening, emulsifying and as a gelling agent [108,117]. Moreover, WPC is also used to improve the gel properties of surimi by inhibiting proteolysis or acting as a gel filler [117]. Shi et al. [118] reported that the combination of 5% WPC and CaCl_2_ 15–18 mmol/kg with a setting time of 60 min enhanced the gel strength of silver carp surimi.

In addition to protein additives, fish mince from marine fish species (white croaker and anchovy) or chicken have been mixed with the mince from carp. Those results showed that the gel mixture could enhance the gel properties of silver carp surimi when blended with 20% white croaker, 10% anchovy, and 50% chicken [119,120,121]. 

#### 5.4.3. Microbial Transglutaminase (MTGase)

TGase is an important enzyme in reconstructed muscle proteins because MHC undergoes the polymerization of proteins induced by endogenous TGase during setting [122]. Various living tissues, such as from microorganisms, vertebrates, invertebrates, and plants, possess endogenous TGase [123]. Microbial transglutaminase (MTGase) has been widely utilized to induce polymerization for forming a gel network [122]. MTGase is mainly found in *Streptoverticillium ladakanum*, and *Streptoverticillium mobaraense* [124]. MTGase catalyzes the acyl-transfer reaction between the carboxamide group of peptide-bound glutamine and a primary amine, and forms a covalent bond, which strengthens the surimi gel network [125]. MTGase has been incorporated into surimi gels from various dark and white-fleshed fish, such as mackerel, lizardfish, etc. [126]. Fang et al. [127] investigated the relationship between cross-linking catalyzed silver carp surimi induced by an MTGase addition (15 U/g protein) on digestibility by pepsin. The addition of the MTG enhanced the gel properties (hardness, chewiness, resilience, breaking force, and deformation), and cross-linking of the surimi, which was indicated by a non-digestibility during the in vitro digestion process. This was mainly due to the higher cross-linking of myofibrillar proteins induced by the MTGase, which reduced the accessibility of pepsin to the hydrolytic site. Guo et al. [128] studied the effects of various setting conditions (40 °C/60 min; 90 °C/30 min; 40 °C/60 min + 90 °C/30 min) in combination with an MTGase addition on the gel properties of vacuum-freeze-dried silver carp surimi. The results showed that the setting condition (40 °C) combination with an MTGase addition (0.06% paste) could induce the formation of MHC cross-linking as indicated by an improved color and increased breaking force. An et al. [129] investigated the application of MTGase on silver carp surimi during short-term frozen storage at −18 °C, and the gelation induced by the MTGase. They reported the unfolding of the myosin structure in the early days of storage, which promoted cross-linking reactions and intermolecular hydrophobic interactions. Furthermore, a Ca^2+^ ion is required for MTGase activity to promote the cross-linking of the gel [30]. An increase in the degree of cross-linking also promotes the digestion of the myosin head, but it does not affect the nutrient value of the surimi gel [130].

#### 5.4.4. Salts

Salts have been widely used to extract myofibrillar proteins and to develop the texture of surimi during cooking [131]. The addition of a neutral salt could increase the hydration capacity and binding properties of the myofibrillar protein which induces the interactions of protein–protein molecules to be more stabilized. Sodium chloride (NaCl) is widely used to produce surimi products [73]. Wang et al. [132] investigated the effects of different concentrations of NaCl (0.1–3.0 M) on the relationship between myosin self-assembly and the physicochemical properties during setting at 4 °C. The results showed that the myosin assembled into filaments by rod–rod ionic linkages and exhibited higher physicochemical properties at low concentrations of NaCl between 1.0–3.0 M; however, a high salt intake could induce hypertension and cardiovascular diseases in consumers [133]. Therefore, to avoid a high sodium intake in food products, alternative salts such as KCl, CaCl_2_, and MgCl_2_ have been used to replace NaCl [134]. Feng al. [135] investigated the effects of the replacement of NaCl with KCl, MgCl_2_ and CaCl_2_ on the textural and rheological properties and microstructure of grass carp surimi. The results showed that the efficiency ranking of chlorinated salts in the gelation of myofibrillar protein was KCl > MgCl_2_ > CaCl_2_ at the same concentration of chlorine ions (0.6 mol/l, pH 7.0). Yu et al. [136] also reported that the physicochemical properties of silver carp surimi gels prepared with KCl showed a better water-holding capacity than gels with a CaCl_2_ addition at the same corresponding ionic strength (0.51, 0.34 and 0.17).

#### 5.4.5. Cryoprotectants

Cryoprotectants are a series of reagents that cooperate with myofibrillar proteins and are used to prevent the denaturation and oxidation of surimi proteins during the frozen storage of surimi. For example, the muscles could be damaged by ice crystal formation during frozen storage [137,138]. Xie et al. [139] investigated the effect of freeze duration on protein denaturation in grass carp surimi by tri-step spectroscopy. The spectral curve-fitting analysis divided the denaturation mechanism of surimi during frozen storage into three periods: a stable period (0–4 weeks), a slow changes period (4–12 weeks), and a deterioration period (>12 weeks). Numerous studies have reported that the addition of cryoprotectants (sucrose and sorbitol mixtures) to surimi gel alleviates the protein freezing denaturation, reduces a hydrophobic group on the protein surface, and prevents ice crystal formation [140,141,142]. Moreover, the gel formation ability and gel quality of surimi are also enhanced by the addition of cryoprotectants [141]. Pan et al. [143] showed that the addition of 6% trehalose in combination with 0.3% polyphosphate had a higher protective efficiency for muscle protein. The result was confirmed by an increase in the salt extractable protein content, Ca^2+^-ATPase activity, and sulfhydryl content during frozen storage. Moreover, polyphosphate could promote the protective effect of the trehalose or sorbitol/sucrose. In addition, various plant or animal protein hydrolysates have been used as a cryoprotectant [144,145]. Protein hydrolysates are generally composed of 2–16 amino acids and their antioxidant activity could depend on the amino acid composition, sequence, as well as the chain length of the peptide or hydrolysate [146]. The addition of fish protein hydrolysates could prevent the denaturation of myofibrillar proteins and increase the unfrozen water of products [147]. Generally, the cryoprotective activity of proteins or peptides results from two major effects, including freezing hysteresis, which is a protection from freezing, and the inhibition of recrystallization, in which organisms probably protect from freezing damages [148]. Both effects are direct results of the immobilization of solid–liquid interfaces in partially frozen treatments [148,149]. Zhang et al. [144] also reported that the addition of a 2% sucrose combination with surimi by-product hydrolysate, prepared by trypsin and alcalase on silver carp surimi, improved the initial gelation properties as well as the water-holding capacity of the gel. Lin et al. [138] investigated the cryoprotective effects of protein hydrolysates from bighead carp grill on silver carp surimi during frozen storage at −18 °C. The results showed that 1% and 2% gill protein hydrolysates had lower oxidation and quality loss than surimi without the treatments. The result was indicated by sulfhydryl, salt-soluble protein concentrations, Ca^2+^-ATPase activity, disulfide bonds, carbonyls, and hydrophobicity. Moreover, the addition of gill protein hydrolysates enhanced the gel strength and textural properties (hardness, springiness, gumminess, chewiness) of the surimi gel.

#### 5.4.6. Other Food Additives

In addition to polysaccharides, proteins, various polyphenols and oils have been incorporated into surimi and surimi products to improve their quality under different conditions.

Polyphenols have been found on the parts of a plant such as fruits, seeds, and leaves, which possess excellent antioxidant activities as well as antibacterial properties [73]. Polyphenols from plant extracts such as tea polyphenols, epigallocatechin gallate (EGCG), and young apple polyphenols were used to improve gel properties and to prevent the oxidation of myofibrillar protein in Asian carp surimi [150,151,152]. For example, Sun et al. [152] reported that the addition of 0.10% young apple polyphenols retarded the lipid oxidation and degradation of soluble myofibrillar protein of grass carp surimi during storage at 4 °C for 7 days. This was confirmed by a lower increase in peroxide value and thiobarbituric acid reactive substances during storage. Moreover, the gel with polyphenol added maintained its color and prevented the degradation of the soluble myofibrillar protein, which was indicated by protein function including the emulsifying activity, emulsifying stability, and surface hydrophobicity.

Oils have been widely used as a texture modifier, color enhancer, or processing aid for surimi production [108]. Numbers of studies have reported the addition of oils such as peanut oil, soybean oil, and fish oil in silver carp surimi [63,153,154]; however, excessive oil contents could reduce the breaking force due to an interference in the formation of a gel network. Furthermore, the replacement of oil for water may enhance the protein concentration in the matrix of a gel [153,154].

**Table 2:** The functional ingredients and their optimum conditions to enhance the quality of surimi and surimi products.

Food Additives	Name of Fish Species	Experimental Conditions	Analysis	Optimum Amount/Treatment Conditions	Reference
Carbohydrate					
Potato starch, corn starch	Silver carp	8% potato starch (modified starch, native starch), and 8% corn starch	TPA, penetration force, gel strength, color evaluation, microstructure, and paraffin section	8% potato starches	[87]
Wheat flour	Silver carp	0%, 10%, 20%, and 30% of surimi combination with wheat flour, setting using microwave heating at 2450 MHz	Temperature distribution, and DSC	10% and 30% of surimi	[91]
Modified starch	Silver carp	Acetic acid esterification starch (AAES), cross-linked esterification starch (CES), cross-linked hydroxypropylated starch (CHS), hydroxypropylated starch (HS), and sorbital combination with sucrose (1:1; wt), storage conditions; temperature −20 °C for 90 days	Gel Strength, Ca^2+^–ATPase activity, SSP content, and SH content	AAES and HS	[95]
Konjac glucomannan (KGM)	Grass carp	0%, 0.5%, 1%, 1.5%, and 2% KGM, storage conditions; temperature −18 °C for 30 days	TPA, WHC, whiteness, SEP content, Ca^2+^ ATPase activity, SH content, and active sulphydryl content	1% KGM	[97]
Starch gum	Silver carp	1.37% hydroxypropylated cassava starch (HCS), 0.44% curdlan (CD), 0.22% κ-carrageenan (KC), and mixtures of 1.37% hydroxypropylated cassava starch, 0.44% curdlan and 0.22% κ-carrageenan (HCK)	Gel strength, TPA, WHC, whiteness, soluble protein content, SEM, raman spectroscopy, and sensory analysis	HCK	[98]
Pectin gum	Silver carp	1% pectin gum and 0.2% CaCl_2_ with different degree of methoxylation, 27–33%, 27–33%, 60%, 65%, 69–72%, and 72%	Torsion test, TPA, and expressible water	Low methoxyl (27–33%)	[99]
Apple pectin, KGM	Silver carp	0.025%, 0.05%, 0.075%, and 0.1% apple pectin combined with 1%, 1.5%, 2%, and 2.5% KGM	Gel strength, TPA, WHC, whiteness, SDS–PAGE, and soluble protein	0.025% apple pectin combined with 2% KGM	[100]
Chitosan	Silver carp	Chitosan with molecular weight (MW), 299, 410, 600, 706, and 880 kDa combination with different degree of deacetylation, 60.5%, 65.4%, 70.8%, 77.3%, and 86.1%	Rheological characteristics, gel strength, WHC, SEM, and molecular forces	Chitosan with MW 880 kDa combination with deacetylation 77.3%	[103]
Nanosized okara	Silver carp	0%, 0.1%, 0.2%, 0.4%, 0.6%, and 0.8% microsized okara insoluble dietary fiber (MIDF) and nanosized okara insoluble dietary fiber (NIDF)	TPA, LF-NMR, MRI, light microscopy observation, and FTIR-ATR	0.8% NIDF	[105]
Seaweed	Silver carp	27.7 g/kg *Ulva intestinalis* seaweed powder, and 5 g/kg *U. intestinalis* sulphated powder, storage conditions; temperature −18 °C for 6 mouths	Proximate compositions, cooking yield, pH, instrumental color evaluation, peroxide value, TBARS, sensory evaluation, and TPA	*U. intestinalis* sulphated powder	[106]
Chicory polysaccharide	Silver carp	0%–8% chicory polysaccharide	Sensory evaluation, fuzzy mathematical, factor weight set, hardness, elastic, TVB-N, pH, TBARS, fatty acids, and microbial analysis	3% chicory polysaccharide	[107]
Protein					
Egg white protein	Silver carp	Egg white protein (EWP), and β-cyclodextrin (βCD) mixture, 0%, 2%, 4%, and 6%, storage conditions; temperature −18 °C for 60 days	FI, circular dichroism, dynamic rheological properties, water loss, TPA, and microstructure	6% EWP-βCD	[111]
Soy protein isolate	Silver carp	0%, 10%, 20%, 30%, and 40% soy protein isolate, cooking conditions; direct cooked 85 °C for 30 min, cooked after setting at 30 °C for 60 min, cooked after 40 °C for 60 min, and cooked after 50 °C for 60 min	Gel strength, breaking force, and breaking distance	10% soy protein isolate cooked after setting at 50 °C for 60 min	[113]
Soy protein isolate	Bighead carp	0%, 10%, 20%, 30%, and 40% soy protein isolate, setting conditions; 30 °C, 35 °C, 40 °C, 45 °C, and 50 °C for 30, 60, and 120 min	Gel strength, and microstructure	10% soy protein isolate with setting condition at 35 °C to 40 °C for 60 min	[116]
Whey protein concentrate (WPC)	Silver carp	1–9% WPC combinate with 1–59 mmol/kg CaCl_2_ setting at 30–90 min	Gel strength, and bending test	5% WPC and 15–18 mmol/kg CaCl_2_ with setting at 60 min	[118]
Microbial transglutaminase (MTGase)	Silver carp	0 and 15 U/g MTGase, digestion time; 0, 5, 30 and 120 min	Extent of cross-linking, TPA, dry matter digestibility, particle size, microstructure, tricine-SDS-PAGE, amino acid composition, and LC-MS	Digestion at 30 min	[127]
MTGase	Silver carp	0 and 9 U/g MTGase, storage condition; temperature −18 °C for 0, 1, 3, 5, 7, 10, and 15 days	TPA, WHC, extent of cross-linking, free amino concentration, TGase activity, SH content, disulfide bond, S0, turbidity, and CD spectrum	5–7 day for promoting the cross-linking	[129]
Salts					
NaCl	Silver carp	0.1, 0.2, 0.3, 0.4, 0.6, 1, 2, and 3 M NaCl, setting conditions; temperature 4 °C	Confocal laser scanning microscopy, UV absorption spectra, Ca^2+^-ATPase activity, S_0_, SH content, reactive sulfhydryl, turbidity, solubility, and particle size	1–3 M NaCl	[132]
NaCl, KCl, MgCl_2_, CaCl_2_	Grass carp	The same concentrate (0.6 mol/L) of NaCl, KCl, MgCl_2_, and CaCl_2_ with 0.1 g/100 mL MTGase, setting conditions; temperature 4 °C	WHC, color evaluation, gel strength, dynamic rheological, microstructure, and raman spectrum	KCl > MgCl_2_ > CaCl_2_	[135]
NaCl, KCl, CaCl_2_	Silver carp	NaCl, KCl, and CaCl_2_ at corresponding to ionic strength of 0.51, 0.34, and 0.17	TPA, WHC, gel strength, chemical bonds, rheological analysis, SH content, solubility, and SEM	KCl	[136]
Cryoprotectants					
Sucrose, sorbitol, trehalose, polyphosphate	Grass carp	4% sucrose + 4% sorbitol, 4% sucrose + 4% sorbitol + 0.3% polyphosphate, 6% trehalose, and 6% trehalose + 0.3% polyphosphate, storage conditions; temperature −18 °C for up to 25 weeks	Ca^2+^-ATPase activity, SH content, SEP content, and gel-forming ability	6% trehalose, and 0.3% polyphosphate	[143]
Protein hydrolysate	Silver carp	4% sucrose (S), 2% surimi by-products hydrolysate by trypsin + 2% sucrose (TS), and 2% surimi by-products hydrolysate by alcalase + 2% sucrose (AS), storage conditions; temperature −18 °C for 3 months	MW distribution, degree of hydrolysis, ABTS radical scavenging activity, reducing power, Fe^2+^-chelating activity, SSP, SH content, carbonyls concentration, Ca^2+^-ATPase activity, fluorescence intensity, S_0_, gel strength, TPA, water distribution, and expressible water content	TS and AS	[144]
Protein hydrolysate	Bighead carp	1% hydrolysate from neutral protease, 2% hydrolysate from neutral protease, and 4% sucrose, storage conditions; temperature −18 °C for 4 months	MW distribution, DPPH radical scavenging activity, chelating activity, SH content, disulfide bonds, carbonyl concentration, Ca^2+^-ATPase activity, SSP, S_0_, gel strength, TPA, and LC-MS	1% and 2% hydrolysates by neutral protease	[138]
Polyphenols					
Tea polyphenols	Grass carp	0, 5, 10, 20, 50, and 100 μmol/g tea polyphenols	Amino acid side-chain groups, raman spectra, S_0_, SDS–PAGE, gel strength, TPA, particle size, and turbiscan stability index	5 and 10 μmol/g tea polyphenols	[150]
Young apple polyphenols	Grass carp	0%, 0.05%, and 0.10% young apple polyphenols, storage conditions; temperature at 4 °C for 7 days	TBARs, TVB-N, PV, color evaluation, soluble myofibrillar protein content, SDS-PAGE, emulsifying activity, emulsifying stability index, S_0_, gel strength, TPA, and sensory evaluation	0.10% young apple polyphenols	[152]
Oil					
Vegetable oils	Silver carp	10, 20, 30, 40, and 50 g/kg of soybean oil, peanut oil, corn oil, and rap oil	Punch test, expressible water, color evaluation, dynamic rheological, transmission electron microscopy, and sensory evaluation	10% peanut oil	[153]
Soybean oils	Silver carp	0%, 1%, 2%, 3%, 4%, and 5% soybean oils	Punch test, TPA, and color evaluation	<3% soybean oil	[154]
Fish oils	Silver carp	0, 3, 6, 9, and 12% fish oils, heating condition; under two-step water bath heating (40 °C for 30 min and 90 °C for 20 min), and water bath-microwave heating (40 °C for 30 min and power intensity 5 w/g for 96 s)	TPA, color evaluation, expressible moisture content, SEM, LF-NMR, and lipid oxidation	6% fish oil, under water bath-microwave heating	[63]

## 6. Challenges

### 6.1. Inferior Gel Forming Ability

Asian carp species are known to have a relatively low gel-forming ability. Luo et al. [155] have completed a study on the gel-forming ability of Asian carp species (common carp, grass carp, and silver carp) compared to that of an Alaska pollock, the marine fish species commonly used for surimi production. The result of this study showed that the Asian carp species had a lower gel-forming ability than an Alaska pollock; however, the Asian carp species still had enough gel-forming ability to be utilized in surimi production [155]. The gel-forming ability of the muscle protein can be affected by several factors such as the muscle sources, protein concentration, heating rate, and heating time. Protein plays an important role in the determining of gel properties [2] and the protein muscle sources can influence the gel-forming ability [2]. Chan et al. [156] reported that the differences in gel-forming abilities of three fish species (cod, herring, and silver hake) were related to the cross-linking abilities of the myosin helical tail. Meanwhile, a number of studies compared the network structure between marine fish and freshwater fish by using microstructure [157,158]. Riebroy et al. [159] reported that marine fish (Atlantic cod) myosin obtained a higher interconnected and finer network structure than freshwater fish (burbot) myosin. The gel-forming ability was increased due to higher myofibrillar protein concentrations [159]. Asian carp species have white flesh which on average is composed of 16.65% protein content [10], while marine fish such as Alaska pollock flesh is composed of 17.5% proteins [160]. A strong and orderly 3-dimensional surimi gel structure can be achieved when surimi gelation occurs under a slow heating rate and a previous study showed that Asian carp species (common carp, grass carp, and silver carp) required a higher temperature and longer duration for surimi gelation compared with Alaska pollock [155]. 

Although Asian carp species have a relatively low gel-forming ability compared to marine fish species, they are usable in surimi manufacturing and their low gel-forming ability can be improved by using various additives such as pectin, curdlan, κ-carrageenan, gelatin, and starch [11,98,99]. Moreover, this gel-forming ability is sufficient for surimi processing and its products.

### 6.2. Muddy Odor

Off-odors and off-flavor represent a significant problem for fish and their products, which affects consumer acceptance [161]. The compounds of off-odors are generally derived from enzymatic reactions, microbial activity, lipid oxidation, and environmental or thermal reactions [162]. The off-odors of fish are mainly caused by the odor components of alcohols, aldehydes, and ketones such as hexanal, nonanal, 1-octen-3-ol and 2, 4-heptadienal (E, E) [163]. In addition, the muddy odor or taste in fish is also considered as one of the major problems in carps or other aquaculture fishes. Generally, geosmin and 2-methylisoborneol (MIB) are the main causes of an earthy or muddy odor, which is produced by cyanobacteria and actinomyces [161]. The accumulation of a muddy odor is also dependent on management practices and water quality [164]. On the other hand, a fishy odor is generally related to diatom, chrysophyte, cryptophyte and dinoflagellate. The polyunsaturated fatty acids (PUFAs) present in those algae could result in the production of unpleasant odors [165]. In addition, the fishy odor of fish is also directly related to lipid oxidation. Due to their high content of PUFAs, the lipids of fish muscle are more prone to oxidation than in other animals. The lipid oxidation is mainly catalyzed by heme proteins and irons, as well as lipoxygenase [166]. Fu et al. [167] reported that the lipid oxidation and fishy odor of silver carp muscle were caused by 12-lipoxygenase. 

Common methods have been used to remove the off-odor in aquatic products such as adsorption, microcapsules, fermentation, and removal by antioxidants [168],but the removal of off-odor is also dependent on deodorizing agents. Thus, the deodorizing agents should be safe and have a mild odor [169]. The deodorizing of surimi products generally involves washing with saline agents. Currently, several methods have been utilized to remove or reduce the off-odor of Asian carp surimi such as ozone treatments and yeast glucan additions [61,170]. 

## 7. Future Directions and Opportunities

Apart from the utilization of Asian carp to substitute for less available marine fish as mentioned earlier, the utilization of Asian carp as a raw material for surimi manufacturing can contribute to the Asian carp problem in the United States as well. Asian carp species, namely, grass carp, silver carp, bighead carp, and black carp are considered as invasive species in the U.S. Their fast reproduction and growth rate are desirable characteristics if they are raised for commercial use but they have no natural predators, making them a threat to native species and the environment. The U.S. government has expended significant effort in attempting to control Asian carp numbers and in preventing their further spread. Using Asian carp as an alternative to marine fish in surimi processing can increase the consumption of Asian carp and lead to a decline in the Asian carp population; however, various things must be undertaken in order to make this strategy work. 

First, the customers’ views on Asian carp must be changed. Currently, Asian carp in the U.S. have a bad reputation and people often view Asian carp as a dangerous, inedible and invasive species. Moreover, its off-flavors and intermuscular bones have also made them unappealing. Consumers or farmers should be educated about the nutritive values as well as their cultural practices to control the Asian carp population growth. According to Li et al. [10], more than 60% of American consumers are willing to purchase products from Asian carp after being informed about their benefits. Processing Asian carp into surimi will remove the intermuscular bones and off-flavor, changing the Asian carp into a more appealing and convenient product. 

Moreover, there are several other challenges for the production of Asian carp surimi in the US. Li et al. [10] suggested that four reasons have made Asian carp surimi in the U.S. currently unsuccessful. Those reasons are (1) Asian carp is not abundant enough, (2) catching Asian carp has a higher risk, (3) the price of Asian carp is not competitive to other fish, and (4) a lack of skilled workers. The problems with the abundancy and price of Asian carp exist because Asian carp are yet to be raised and produced as a commercial fish. For skilled personnel, experienced personnel from countries familiar with handling Asian carp could be hired from Asian countries, such as China. Asian carp are important freshwater fish species in China and in 2020, the production of Asian carp in China reached 13 million tons. The consumption demand for surimi products in China is increasing, with the production volume of surimi products in China recorded at around 1.3 million tons in 2020 [171]. Asian carp have also been used to produce a variety of surimi products in China, such as fish balls, fish tofu and fish cakes. Due to this increasing demand for surimi products in China, there is pressure increasing for the production of surimi material resources, especially Asian carp. Currently, there is huge progress in the development of aquaculture technology, such as new feeding techniques, breeding methods, and farm management practices [172,173,174], which have led to increases in the production of Asian carp. Therefore, Asian carp have the potential to be produced in far greater numbers than marine fish. 

As the demand for Asian carp in the U.S. grows and Asian carp begins to be raised for consumption, their reputation will be changed from being a dangerous, inedible and invasive species to a nutrient-rich and easy-to-produce commercial fish.

## 8. Conclusions

Asian carps have a great potential in surimi manufacturing. Their great abundance, appealing white flesh and decent gel-forming ability have made them viable alternatives to marine fish, which are currently used in surimi production. In this review, we have summarized the challenges faced in the production of Asian carp surimi along with solutions to improve their quality. The utilization of Asian carp in surimi production also can contribute to solving the threat from Asian carp in the U.S. as well.

## Data Availability

Not applicable.

## Abbreviations

TPA	Textural profile analysis
SDS-PAGE	Sodium dodecyl sulfate-polyacrylamide gel electrophoresis
SEM	Scanning electron microscopy
WHC	Water holding capacity
LF-NMR	Low-frequency nuclear magnetic resonance
SH	Sulfhydryl
DSC	Differential scanning calorimeter
SSP	Salt-soluble protein
SEP	Salt extractable protein
MRI	Magnetic resonance imaging
FTIR-ATR	Fourier transform infrared attenuated total reflection spectroscopy
TBARS	Thiobarbituric acid reactive substances
TVB-N	Total volatile basic nitrogen
FI	Fluorescence intensity
LC-MS	Liquid chromatography-mass spectrometry
CD	Circular dichroism
S_0_	Surface hydrophobicity
MW	Molecular weight
ABTS	2, 2-azinobis (3-ethylbenzothiazoline-6-sulfonic acid) diammonium salt
DPPH	1,1-Diphenyl-2-picrylhydrazyl
PV	Peroxide value
